# Functional performance and 30-day postoperative mortality after emergency laparotomy—a retrospective, multicenter, observational cohort study of 1084 patients

**DOI:** 10.1186/s13741-020-00143-7

**Published:** 2020-05-05

**Authors:** Mirjana Cihoric, Line Toft Tengberg, Nicolai Bang Foss, Ismail Gögenur, Mai-Britt Tolstrup, Morten Bay-Nielsen

**Affiliations:** 1 Department of Anaesthesiology and Intensive Care Medicine, Hvidovre University Hospital, Hvidovre, Kettegaard allé 30, 2650 Hvidovre, Copenhagen, Denmark; 2 Department of Surgery, Center for Surgical Science, Zealand University Hospital, Koege, Denmark; 3 Department of Gastrointestinal Surgery, Copenhagen University Hospital, Herlev, Copenhagen, Denmark; 4 Department of Gastrointestinal Surgery, Hvidovre University Hospital, Copenhagen, Denmark

**Keywords:** Emergency laparotomy, Frailty, 30-day mortality

## Abstract

**Background:**

Despite the importance of predicting adverse postoperative outcomes, functional performance status as a proxy for frailty has not been systematically evaluated in emergency abdominal surgery. Our aim was to evaluate if the Eastern Cooperative Oncology Group (ECOG) performance score was independently associated with mortality following high-risk emergency abdominal surgery, in a multicentre, retrospective, observational study of a consecutive cohort.

**Methods:**

All patients aged 18 or above undergoing high-risk emergency laparotomy or laparoscopy from four emergency surgical centres in the Capitol Region of Denmark, from January 1 to December 31, 2012, were included. Demographics, preoperative status, ECOG performance score, mortality, and surgical characteristics were registered. The association of frailty with postoperative mortality was evaluated using multiple regression models. Likelihood ratio test was applied for goodness of fit.

**Results:**

In total, 1084 patients were included in the cohort; unadjusted 30-day mortality was 20.2%. ECOG performance score was independently associated with 30-day mortality. Odds ratio for mortality was 1.70 (95% CI (1.0, 2.9)) in patients with ECOG performance score of 1, compared with 5.90 (95% CI (1.8, 19.0)) in patients with ECOG performance score of 4 (*p* < 0.01). Likelihood ratio test suggests improvement in fit of logistic regression modelling of 30-day postoperative mortality when including ECOG performance score as an explanatory variable.

**Conclusions:**

This study found ECOG performance score to be independently associated with the postoperative 30-day mortality among patients undergoing high-risk emergency laparotomy. The utility of including functional performance in a preoperative risk assessment model of emergency laparotomy should be evaluated.

## Introduction

Continued increase in the number of emergency surgical procedures performed in the elderly is seen as a result of rapid expansion of the aging population, with the 65 and over share due to reach 1.6 billion by 2050 (Wang et al. 2016; He et al. 2016).

Given that emergency laparotomy is associated with a substantial degree of morbidity, 30-day mortality above 20 percent (Saunders et al. 2012; Tengberg et al. 2017a; Vester-Andersen et al. 2014) and loss of quality of life (Tolstrup et al. 2017), and accurate identification of patients who are unlikely to benefit from surgical intervention as well as those requiring more extensive observation and optimization is essential.

Dissimilarities in outcomes among elderly patients have heightened awareness that measures of performance status and function, other than chronological age alone, are important predictors of surgical outcomes (Saxton and Velanovich 2011; Gilbert et al. 2017).

Several studies identify frailty as an independent risk factor for major morbidity, mortality, protracted length of stay, and institutional discharge in elective surgical care, but also increasingly in acute surgical care (Tolstrup et al. 2017; Saxton and Velanovich 2011; Kim et al. 2014; Kenig et al. 2015; Makary et al. 2010; Hewitt et al. 2015), though with limited studies focusing solely on emergency laparotomy patients (Hamidi et al. 2018; Joseph et al. 2016; Lorenzon et al. 2017; Mogal et al. 2017; Akyar et al. 2018; Seib et al. 2018).

While significant, frailty assessments used in these studies are comprehensive and thereby potentially time-consuming, e.g., The Modified Frailty Index (mFI) score (Panayi et al. 2018), containing 11–20 variables depending on which model is used, and Frailty Index(FI) containing over 30 variables (Joseph et al. 2016; Seib et al. 2018). A most recent study assessing frailty by Clinical frailty Scale (Parmar et al. 2019) did, however, show clear feasibility and quickness when applied to emergency laparotomy.

Functional performance has, in some studies, been indicative of poor postoperative rehabilitation (Kristensen et al. 2010; Jønsson et al. 2017), and evaluation of physical performance is considered one of the cornerstones in defining frailty (Lytwyn et al. 2017).

Based on the excellent prognostic performance of Eastern Cooperative Oncology Group Performance Status (PS) (Schiller et al. 2002), a simple physician-rated functional performance scale, the in elective patient population, the aim of this study was to evaluate if the ECOG performance Score, was independently associated with postoperative mortality following major emergency abdominal surgery, adding further to the body of knowledge and options for assessment of functional performance as a proxy for frailty.

## Methods

Approval was given by the Danish Data Protection Agency and the Danish Health and Medicines Authority (207-58-0015). In consensus with the Danish law, The Regional Committee on Health Research Ethics waived the requirement for informed patient consent (H-3-2013-078).

The manuscript was prepared according to the Strengthening the Reporting of Observational Studies in Epidemiology (STROBE) statement (von Elm et al. 2008).

This study was a multicenter, retrospective, observational study of a consecutive cohort. The data was collected on all patients aged 18 or above undergoing high-risk emergency abdominal surgery, defined as immediate emergency laparoscopy or laparotomy due to intestinal obstruction, intestinal perforation, intestinal ischemia, or intra-abdominal bleeding, from four emergency surgical centers in the Capital Region of Denmark (covering the care of 1.62 million inhabitants), from January 1 to December 31, 2012. Definition of high-risk emergency abdominal surgery did not differ in the respective centers.

Included were all patients under suspicion of abdominal pathology requiring immediate emergency laparoscopy or laparotomy, including reoperations after elective gastrointestinal surgery. Patients with the following procedures were excluded: appendectomies, cholecystectomies, negative diagnostic laparoscopies/laparotomies, herniotomies without bowel resections, sub-acute internal hernias after gastric bypass surgery, sub-acute surgery for inflammatory bowel diseases, reoperation owing to fascial separation with no other abdominal pathology identified, and sub-acute colorectal cancer-surgery were excluded from the cohort. Sub-acute surgery was defined as surgery planned within 48 h. Traumas, gynecological, urogenital, and other vascular pathology were also excluded, as were pregnant patients.

Surgeries were categorized as either primary laparoscopy/laparotomy or reoperations for acute complications after elective surgery. Reoperations within 30 days postoperatively after initial emergency laparoscopy/laparotomy was considered a complication and were not included.

Baseline patient characteristics were retrieved from electronic patient records, by systematically screening all patients entered in the electronic operation booking system used in all participating hospitals. Written patient records were reviewed manually for a 30-day postoperative period to identify possible complications. The surgical procedure was linked to the Danish Anaesthesia Database and the Danish Civil Registration System, thereby ensuring a hundred percent follow-up on mortality through the patients’ social security number.

Data collected was age, sex, American Society of Anaesthesiologist (ASA) score (ranging from 0 (lowest risk) to 5 (highest risk), serum albumin, existence of comorbidity, indication for surgery (dichotomized in intestinal obstruction and all other indications, surgical characteristics (primary/re-operation, laparotomy/laparoscopy, and specific procedure), patient performance status using the Eastern Cooperative Oncology Group performance score, and preoperative sepsis score (0, no sepsis; 1, systemic inflammatory response syndrome (SIRS); 2, sepsis; 3, severe sepsis; 4, septic shock).

Data gathered on sepsis was done according to 2012 sepsis guidelines (Dellinger et al. 2013). In 2017, a redefinition of the guidelines was published, with the introduction of quick SOFA (qSOFA—Sepsis Related Organ Failure Assessment) score for identification of patients with heightened risk of mortality if meeting ≥ 2 of the following criteria: respiratory rate of 22/min or greater, altered mentation, or systolic blood pressure of 100 mmHg or less (Rhodes et al. 2017; Singer et al. 2016). This meant that SIRS criteria were no longer used to define sepsis and septic shock. We took this under consideration when evaluating data and divided the patients in two groups, sepsis score < 2 and ≥ 2 when applying to logistic regression models, i.e., division between sepsis vs severe sepsis/septic shock.

In this study, frailty was assessed using the ECOG performance score as a proxy, now part of the ECOG-ACRIN Cancer Research Group (Oken et al. 1982; Buccheri et al. 1996).

**Fig. 1:**
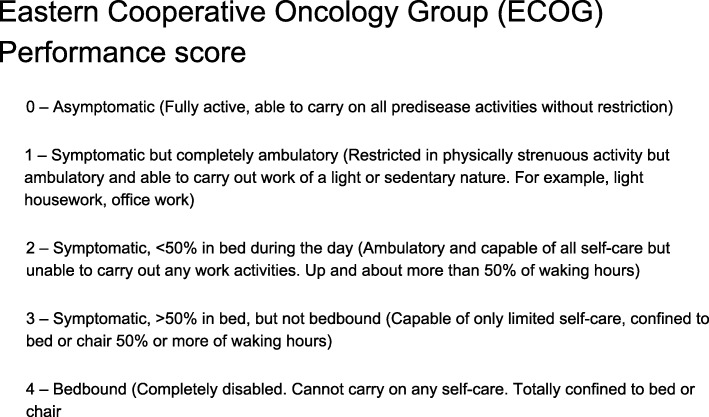
Eastern Cooperative Oncology Group (ECOG) Performance score

The score describes a patient’s level of functioning in terms of their ability to care for themselves, daily activity, and physical ability (walking, working, etc.) and ranges from 0 to 4 (Vester-Andersen et al. 2014), with 0 = describing perfect health (fully active, unrestricted) and 4 = completely disabled and cannot carry on any self-care, totally confined to bed or chair (Fig. 1).

The performance score was assigned retrospectively by a total of six clinicians from the four surgical centers, based on all available information from the medical records which included systematic recording of patient premorbidity performance and need for assistance, and incorporating these according to the ECOG performance score model.

All data collectors underwent educational training sessions to ensure that both the assessment of the performance status and complications were recorded in a standard manner using the ECOG performance score and Clavien–Dindo classification respectively. We evaluated the consistency of these recording accuracies in a random sample of 30 patients and found no important differences in assessment or classifications.

### Study outcome

Primary outcome was an independent association between ECOG performance score and postoperative 30-day mortality. Updated mortality data was extracted January 1, 2015.

### Statistical analysis

Descriptive analysis was carried out for the entire study population. Candidate variables for mortality prediction were selected based on findings in the available literature, as well as clinical experience.

Candidate variables for inclusion in the final multivariate logistic regression model were evaluated by univariate analysis of risk of 30-day mortality, with candidates having *p* value of < 0.25 included in the final model. For univariate analysis, we used the Pearson chi-squared test with Yates’ continuity correction where needed (Table 2).

Pearson’s chi-squared test and Fisher’s exact tests were used for analysis of categorical variables, demographic characteristics, and comorbidities.

Continuous variables were analysed by Mann-Whitney *U* test.

The association of ECOG performance score with 30-day postoperative mortality was evaluated in a logistic regression model, with 30-day postoperative mortality as the dependent variable and ASA, indication for surgery, cardiovascular comorbidity, albumin, presence of sepsis, and ECOG performance score as independent variables. In the model, missing values were substituted by values generated by multiple imputations, with reporting based on pooled coefficients.

Likelihood ratio test was applied, comparing the goodness of fit of two logistic regression models, one with and one without ECOG performance score. Model 1 included ASA, indication for surgery, cardiovascular comorbidity, albumin, and presence of sepsis, while model 2 was added with the ECOG performance score.

The long-term survival of the cohort was illustrated with Kaplan–Meier survival statistics stratified according to the ECOG performance score.

A *p* value of < 0.05 was considered significant.

All analyses were performed using the R statistical software, an open source scripting, data analysis, and graphical environment available without cost for most operating systems (www.r-project.org).

## Results

Baseline characteristics are shown in Table 1. From January 1 to December 31, 2012, 1139 patients met the inclusion criteria. Fifty-five patients were excluded due to the missing ECOG performance score data. Analyses were conducted to identify potential differences between the patients with or without missing data on several predictor variables (data not shown), and none were found.

**Table 1:** Baseline characteristic of patients undergoing emergency high-risk abdominal surgery

Variables	Total (*n* = 1084) (%)
Age; years*	70
18–65	413 (38.1)
66–75	268 (24.7)
76–80	126 (11.6)
81+	277 (26.3)
Female gender	586 (54.1)
**ASA classification**	
1	141 (13.0)
2	454 (41.9)
3	381 (35.2)
4–5	108 (10.0)
Co-morbidities	
Chronic obstructive pulmonary disease	173 (16.0)
Cardiovascular disease	
Hypertension	479 (44.2)
Atrial fibrillation	119 (11.0)
Heart failure	77 (7.1)
Ischemic heart disease**	140 (12.9)
Diabetes requiring medication	105 (9.7)
Stroke	93 (8.6)
Cirrhosis	29( 2.7)
Dialysis dependent renal failure	4 (0.4)
Preoperative sepsis status	
Non infected preoperatively	441 (38.2)
SIRS	15 (1.4)
Sepsis	307 (28.3)
Severe sepsis	46 (4.2)
Septic shock	34 (3.1)
Unknown	268 (24.7)
**Preoperative performance status ECOG**	
0	522 (48.2)
1	313 (28.9)
2	148 (13.7)
3	84 (7.8)
4	17 (1.6)
Surgery characteristics	
Pathology:	
Perforation	431 (39.8)
Obstruction	623 (57.5)
Ischemia	196 (18.1)
Malignancy	211 (19.5)
Type:	
Reoperation after elective surgery	190 (17.5)
Primary	894 (82.5
Procedure	
Laparoscopic surgery	111 (10.2)
Laparoscopic converted to laparotomy	188 (17.3)
Laparotomy	785 (72.5)

Values in parentheses are percentages unless indicated otherwise; *values are median (i.q.r)

*ASA* American Society of Anaesthesiologists, *SIRS* Systemic Inflammatory Response Syndrome, *ECOG* Eastern Cooperative Oncology Group Performance Status Score.

**Previous percutaneous coronary intervention, cardiac surgery, or angina

Data presented in this table has been previously published, though not to this extent or in this context in Tengberg LT, Cihoric M, and Foss NB et al. (2017). Complications after emergency laparotomy beyond the immediate postoperative period—a retrospective, observational cohort study of 1139 patients. Anaesthesia. 72 (Saunders et al. 2012):309–16

The remaining 1084 patients composed our study population. Unadjusted 30-day postoperative mortality was 20.2%.

Five hundred and twenty-two (48%) patients had an ECOG performance score of 0 (normal activity, no restrictions), and 313 (29%) had a score of 1 describing the patients as having symptomatic restrictions in strenuous activity. Twenty-three percent of the cohort had a score of 2 or more, ranging from spending up to 50% of waking hours in bed to being bedbound.

In univariate analysis, increasing age, higher ASA-score, higher ECOG performance score, sepsis, cardiovascular morbidity, low albumin level, and surgery for indications other than obstruction (i.e., ischemia and perforation) were associated with increased risk of mortality (Table 2).

**Table 2:** Association between selected preoperative variables and postoperative Mortality. Univariate analysis

	No. of patients	30-day postoperative mortality (%)	*p* value
Age < 0.0001*			
18–65	413	28 (6.8)	
66–70	159	30 (18.9)	
71–75	133	29 (21.8)	
76–80	127	37 (29.1)	
> 80	252	91 (36.1)	
**Sepsis score**			< 0.0001**
0–2	1004	170 (16.9)	
3–4	80	45 (56.3)	
**ECOG score**			< 0.001*
0	522	36 (6.9)	
1	313	69 (22.0)	
2	148	58( 39.2)	
3	84	41 (48.8)	
4	17	11 (64.7)	
**ASA**			< 0.0001*
1	141	2 (1.4)	
2	454	30 (6.6)	
3	381	115( 30.2)	
4–5	108	68 (63.0)	
**Cardiovascular morbidity**			< 0.0001**
Yes	540	149 (27.6)	
No	544	66 (12.1)	
**Albumin(g/L)**			< 0.0001**
Low (< 36 g/L)	506	148 (29.2)	
Normal (36–48 g/L)	385	45 (11.7)	
High (> 48 g/L)	79	1 (1.2)	
**Indication for surgery**			< 0.0001**
Obstruction	494	68 (13.7)	
Other	550	136 (24.7)	

*Pearson’s chi-squared test, **Pearson’s chi-squared test with Yates’ continuity correction

Values in parentheses are percentages unless indicated otherwise; *values are median (i.q.r). *ECOG* Eastern Cooperative Oncology Group Performance Status Score, *ASA* American Society of Anaesthesiologists. Cardiovascular comorbidity: Hypertension, Atrial fibrillation, Heart failure, previous percutaneous coronary intervention, cardiac surgery, or angina

In a multivariate logistic regression model, ECOG performance score was an independent predictor of 30-day postoperative mortality (*p* < 0.01, LR test). Odds ratio for mortality increased from 1.70 (95% CI [1.0, 2.9]) in patients with ECOG performance score of 1 to 5.90 (95% CI (1.8, 19.0)) in patients with ECOG performance score of 4 (*p* < 0.01) (Table 3).

**Table 3:** Risk factors for 30-day postoperative mortality following emergency high-risk abdominal surgery. Multivariable logistic regression analysis

	OR	95% CI	*p* value
Age	1.0	(1.01, 1.05)	0.0001
ASA 1	1 REF		
ASA 2	1.7	(0.4, 7.7)	0.47
ASA 3	5.6	(1.3, 24.7)	002
ASA 4–5	17.0	(3.8, 79.0)	0.001
Sepsis score 0–2	1 REF		
Sepsis score > 2	2.6	(1.4, 4.7)	0.002
Albumin low (< 34 g/L)	1 REF		
Albumin normal (36–48 g/L)	0.5	(0.3, 0.7)	0.0005
Albumin high (> 48 g/L)	0.1	(0.01,0.9)	0.03
ECOG 0	1 REF		
ECOG 1	1.7	(1.0, 2.9)	0.04
ECOG 2	3.2	(1.78, 5.8)	< 0.0001
ECOG 3	3.9	(1.8, 7.9)	< 0.0001
ECOG 4	5.9	(1.8, 19.0)	0.003
Cardiovascular comorbidity	0.9	(0.6, 1.4)	0.76
Indication for surgery (obstruction vs other)	0.7	(0.6, 1.4)	0.14

*OR* odds ratio, *CI* confidence interval, *ASA* American Society of Anesthesiologists. Cardiovascular comorbidity: hypertension, atrial fibrillation, heart failure, previous percutaneous coronary intervention, cardiac surgery, or angina. *ECOG* Eastern Cooperative oncology Group Performance Status Score

**Fig. 2:**
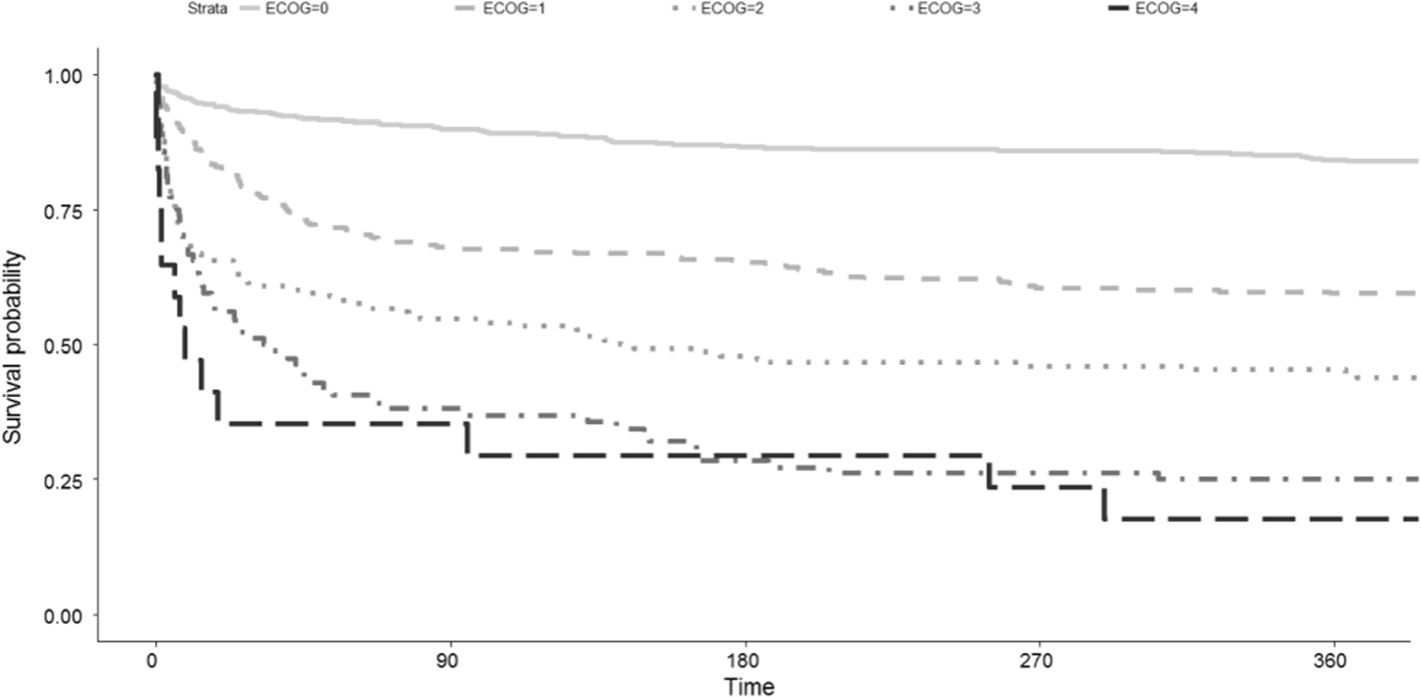
Kaplan–Meier estimated survival within days of acute abdominal surgery stratified according to ECOG performance score

Figure 2 illustrates Kaplan–Meier estimated survival within days of emergency abdominal surgery stratified according to the ECOG performance score. Patients with an ECOG performance score of 0, 1, 2, 3, or 4 had a 12-month mortality rate of 15, 40, 54, 74, and 80% respectively.

**Fig. 3:**
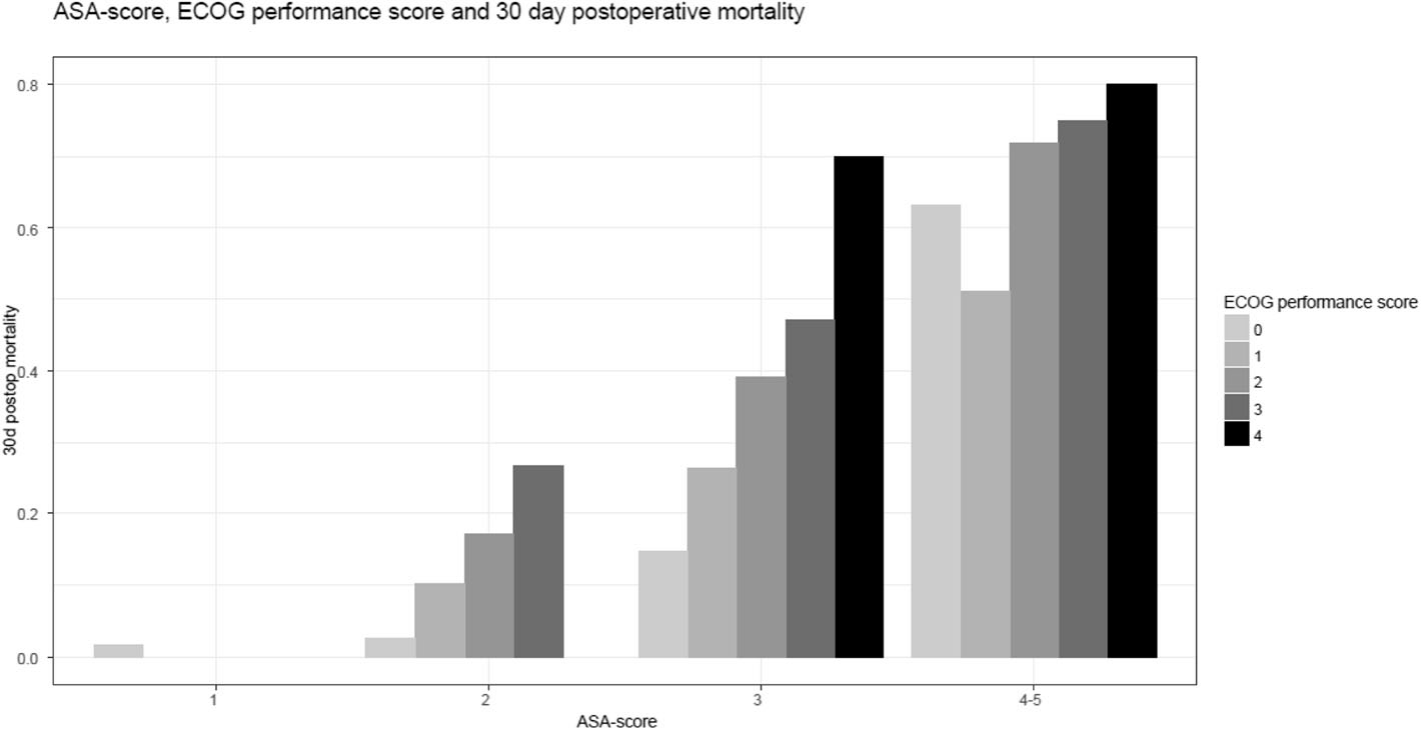
Changes in mortality with increasing ECOG performance score, independently of ASA score

Figure 3 shows the increasing mortality within each ASA group, when stratified on ECOG score.

## Discussion

This study found functional performance to be independently associated with mortality after high-risk emergency laparotomy or laparoscopy. Specifically, the higher the ECOG performance score, the greater the risk of 30-day postoperative mortality.

Older patients represent a growing surgical population without well-defined guidelines for their management. These patients frequently present with inadequate physiological reserve to endure surgery, followed by postoperative strain (Revenig et al. 2013). This lack of reserve is often neglected or approached unsystematically. As other studies have suggested, this tendency is perhaps due to a lack of understanding of geriatric frailty, but even more so, the absence of appropriate methods for evaluating the older population in an emergent care setting. There does not seem to be a consensus on the definition or measurement of frailty (Morley et al. 2013), even in geriatric literature.

Risk assessment and prediction tools exist to help guide clinicians (Vincent and Moreno 2010), one of the most widely used is ASA, a simple but subjective assessment of preoperative risk relating to organ-specific dysfunction, identifying patients in need of a more intensive perioperative care. Other organ-specific-based scores include Physiological and Operative Severity Score for the enUmeration of Mortality and morbidity (POSSUM), Acute Physiology and Chronic Health Evaluation (APACHE II,III, IV), Simplified Acute Physiology Score (SAPS II), and Sequential Organ Failure Assessment (SOFA), primarily used in the Intensive Care Units and previously shown to be complex because of the number of variables needed to complete the score, thus making them difficult to utilize during the emergency course (Oliver et al. 2015; Chandra et al. 2009). Furthermore, tools for critical care were not originally created for perioperative risk assessment, but for comparative audit, and the raw data needed for calculation of risk is not always easily obtainable, particularly outside that of the ICU setting.

It is noteworthy that none of these models incorporate any assessment of overall functional capacity or frailty, as a growing body of evidence points to the importance of frailty as a predictor of outcome, in both elective and emergency care, across a wide range of medical and surgical conditions.

Only few studies regarding frailty and emergency high-risk abdominal surgery exist (Kenig et al. 2015; Hewitt et al. 2015; Parmar et al. 2019; Li et al. 2016). A study in patients undergoing emergency laparotomy found that preoperative P-POSSUM and ASA scoring predicted mortality as moderate discriminators in elderly patients undergoing an emergency laparotomy, and that the addition of frailty scoring in conjunction with P-POSSUM in this high-risk group might better identify those with a high risk of mortality (Sharrock et al. 2017).

A study from 2015 suggested that inclusion of ASA score and ECOG performance score, individually or in combination, improved risk adjustment models after cancer surgery (Young et al. 2015). A study, assessing the impact of frailty on mortality in elderly ICU patients, found that at time of admission to the ICU, the common markers of illness severity (SAPS II and SOFA) did not differ between the frail and non-frail patients (Le Maguet et al. 2014).

A recent study from 2017 (Krinsley et al. 2017), using a three-category score based on the performance of basic daily living activities, found not only that preadmission functionality score was independently associated with mortality among critically ill intensive care patients, but also found frailty score to impact the performance of APACHE IV by impacting the observed vs the predicted mortality percentage, indicating that the APACHE IV model may underestimate risk in patients with impaired functionality score.

A recently developed risk prediction model from the National Emergency Laparotomy Audit (NELA) does not include frailty or level of functioning prior to surgery (Eugene et al. 2018). Interestingly, they do note that the ASA score in itself did not accurately describe 30-day mortality risk in elderly patients, signifying the importance of including other relevant risk factors in risk prediction in emergency laparotomy patients. Our finding of ECOG performance score to be independently associated with 30-day postoperative mortality, and to discriminate mortality within each ASA-group, supports the relevance of including a measure of frailty in models of risk after emergency laparotomy.

Accurate risk prediction is important in the context of scientific comparison and benchmarking across different populations and studies. In a clinical context, it is relevant to inform preoperative discussions and informed consent with patients and families in especially high-risk patients, where palliative care could be a relevant option. A recent study on decision-making in emergency laparotomy found that the decision of whether to perform an emergency laparotomy is complex, with multiple influencing factors (Hendra et al. 2018). The study demonstrates a difference in decision-making and risk attitudes between surgeons; however, premorbid state or frailty was considered an important factor, as important as age, comorbidities, and anaesthetic risk, indication that frailty is a factor to be included/evaluated in risk and prediction models.

This seems to indicate that frail patients require a different approach to care beyond assessing organ-specific dysfunction. There is a need for a more holistic approach which addresses individual needs through a goal-oriented care planning process structured around functional status.

We recognize important limitations of the study. Data collection and identification of the physiological score were done retrospectively, subjecting our findings to potential bias, though mortality was not calculated at the time of data extraction. Retrospective assessment has its weaknesses; however, as ECOG already was implemented in the clinic, in form of description of the functional status during the initial assessment of the patient, an obligatory part of a patient journal, we believe that we had the relevant data on patients’ possible limitations or lack of function in daily life. If the patient was unable to assist in the assessment, either relatives or information from the primary sector was available through electronic patient records. Furthermore, several studies assessing ECOG performance score did so retrospectively (Takahashi et al. 2017; Murakawa et al. 2019). Six clinicians from four different surgical centers collected the data. By following a standardized manual, we sought to minimize the risk of inter-rater disagreement and ensure collection of high-quality data; all data collectors underwent educational training sessions to ensure that the assessment of the performance status was done in a standard manner. Fifty-six patients had missing data, meaning that description of their performance status was not registered in the initial assessment upon hospitalization; this is, however, not as large a group as we might have expected. Analyses were conducted to identify potential differences between the patients with or without missing data on several predictor variables (data not shown), and none were found. However, these findings ought to be confirmed in future prospective trials.

Furthermore, the logistic regression analysis did not take into account the experience of the primary surgeon and anaesthesiologist, as well as timing of the surgery or whether surgery was performed during the night or daytime. This could have had a potential impact on the postoperative outcome. Lastly, data from this study are from 2012*.* Data in this study is older, yes, and the mortality is high. Around that time, a focus shifted on this patient group and several groups, AHA group from Hvidovre, Denmark (Tengberg et al. 2017b), as well as ELPQuiC- group from the UK (Huddart et al. 2015), designed multimodal protocols for improving the outcomes, and these studies have now resulted in awareness and integration of standardized process pathways reducing postoperative mortality to app. 10–18%, depending on study. Our result straddles the time period where these interventions were performed.

The mortality of 9.5 percent in NELA study (NELA study n.d.),is the result of significant focus on optimizing the care pathway in emergency laparotomy. The primary problem in comparing postoperative mortality directly between different nations/surgical cultures is patient selection, which can introduce a selection bias. Patient selection is potentially impacted by professional cultural differences, as demonstrated in comparisons between US and UK cohorts (Markar et al. 2019a; Markar et al. 2019b). This has recently been described in a recent cohort study of the “NoLap” population, which represent data from a single UK centre with 30% of candidates for mergency laparotomy not receiving surgery due to perceived futility, which may impact overall mortality (McIlveen et al. 2019).

Although formal studies in “NoLap” is almost non-existent, the culture in Denmark mandates surgery before assessment of futility. However, there is no data to support this statement.

Another interesting notion, in the NoLap cohort, is that the functional independence was 81% in the group having laparotomy compared to 30% in the group not having surgery, and as such functional assessment is of great importance in triage decisions, although it would seem that it is used somewhat arbitrarily at present.

We also recognize several strengths: this is, to our knowledge, the first study to examine ECOG performance score in an exclusively emergent setting, focusing on emergency high-risk abdominal surgery. Recent studies do examine frailty in emergency general surgery (Joseph et al. 2016; Mogal et al. 2017; Akyar et al. 2018; Panayi et al. 2018), however, the definition of emergency general surgery differs. While the majority of these studies group appendectomies, cholecystectomies and intestinal obstruction, pathophysiology of these conditions cannot be compared. Also, the sample size for ECOG 4–5 was small, and the stratification by ASA could be considered unreliable.

In an emergency setting, comprehensive frailty screening instruments such as Fried Frailty Phenotype (Makary et al. 2010) assessing weight loss, grip strength, exhaustion, physical activity, gait speed, or Canadian Study of Health and Aging Frailty Index (Rockwood et al. 2005) with a count of 70 factors assessing frailty, are probably not feasible due to patients acute condition and time sensitive nature of the emergent case. In studies mentioned above, Modified Frailty Index and Frailty Index was used, and while these frailty assessments are perhaps less subjective, they are more comprehensive.

While functional performance cannot stand alone as a proxy for frailty, it is indicative of a potentially frail individual. Incorporating physical performance measures in evaluation of frailty has shown to be highly predictive of clinical outcome of elderly surgical patients presenting for emergency orthopedic surgery (Gary n.d.; Parker and Palmer 1993; Foss et al. 2006), even more so than ASA. Furthermore, recent study (Parmar et al. 2019) of a large cohort found Rockwood Clinical Frailty Score (CFS) quick and feasible in an emergency laparotomy cohort, a score incorporating functional performance in assessing frailty, much like ECOG performance score. Our study supports these findings, and further underlines the importance of functional performance in patients undergoing emergency laparotomy. Furthermore, this study also incorporates all patients aged 18 or over, stressing the fact that frailty is not only a function of the chronological age; part of the utility of a reliable frailty scale is in identifying younger patients at risk for adverse postoperative outcomes.

Also, this multicentre study contains large homogenous dataset with a 100% follow-up of postoperative mortality.

In conclusion, growing body of evidence supports the notion that preoperative functional performance assessments are feasible and provide critical information in regard to frailty beyond the traditional surgical risk assessments. This study found ECOG performance score to be independently associated with postoperative 30-day mortality among patients undergoing emergency laparotomy.

## Data Availability

The data set used and/or analyzed during current study are readily available from the corresponding author on reasonable request.
